# Moderate Levels of Pre-Treatment HIV-1 Antiretroviral Drug Resistance Detected in the First South African National Survey

**DOI:** 10.1371/journal.pone.0166305

**Published:** 2016-12-01

**Authors:** Kim Steegen, Sergio Carmona, Michelle Bronze, Maria A. Papathanasopoulos, Gert van Zyl, Dominique Goedhals, William MacLeod, Ian Sanne, Wendy S. Stevens

**Affiliations:** 1 Department of Molecular Medicine and Haematology, University of the Witwatersrand, Johannesburg, South Africa; 2 National Health Laboratory Services, Johannesburg, South Africa; 3 Division of Medical Virology, Stellenbosch University, Stellenbosch, South Africa; 4 Department of Medical Microbiology and Virology, University of the Free State, Bloemfontein, South Africa; 5 Center for Global Health and Development, Boston University School of Public Health, Boston, Massachusetts, United States of America; 6 Health Economics and Epidemiology Research Office, Department of Internal Medicine, School of Clinical Medicine, Faculty of Health Sciences, University of the Witwatersrand, Johannesburg, South Africa; 7 Right to Care, Johannesburg, South Africa; University of Cincinnati College of Medicine, UNITED STATES

## Abstract

**Background:**

In order to assess the level of transmitted and/or pre-treatment antiretroviral drug resistance to HIV-1, the World Health Organization (WHO) recommends that regular surveys are conducted. This study’s objective was to assess the frequency of HIV-1 antiretroviral drug resistance in patients initiating antiretroviral treatment (ART) in the public sector throughout South Africa.

**Methods:**

A prospective cross-sectional survey was conducted using probability proportional to size sampling. This method ensured that samples from each province were proportionally collected, based on the number of patients receiving ART in each region. Samples were collected between March 2013 and October 2014. *Pol* sequences were obtained using RT-PCR and Sanger sequencing and submitted to the Stanford Calibrated Population Resistance tool v6.0.

**Results:**

A total of 277 sequences were available for analysis. Most participants were female (58.8%) and the median age was 34 years (IQR: 29–42). The median baseline CD4-count was 149 cells/mm^3^ (IQR: 62–249) and, based on self-reporting, participants had been diagnosed as HIV-positive approximately 44 days prior to sample collection (IQR: 23–179). Subtyping revealed that 98.2% were infected with HIV-1 subtype C. Overall, 25 out of 277 patients presented with ≥1 surveillance drug resistance mutation (SDRM, 9.0%, 95% CI: 6.1–13.0%). Non-nucleoside reverse transcriptase inhibitor (NNRTI) mutations were the most numerous mutations detected (n = 23). Only two patients presented with a protease inhibitor (PI) mutation. In four patients ≥4 SDRMs were detected, which might indicate that these patients were not truly ART-naïve or were infected with a multi-resistant virus.

**Conclusions:**

These results show that the level of antiretroviral drug resistance in ART-naïve South Africans has reached moderate levels, as per the WHO classification. Therefore, regular surveys of pre-treatment drug resistance levels in all regions of South Africa is highly recommended to monitor the changing levels of pre-treatment antiretroviral drug resistance.

## Introduction

South Africa has the largest antiretroviral treatment (ART) program in the world with an estimated cumulative number of individuals on treatment of 3.1 million[1]. Transmitted HIV drug resistance (TDR) implies that newly-infected individuals are infected with a drug-resistant virus. With increased treatment coverage, TDR is expected to increase, especially in settings relying on clinical and CD4 monitoring to detect treatment failure, whereas the early detection of failure through viral load monitoring coupled with a timely switch to second-line therapy may protect against an increase in TDR [2]. The presence of pre-treatment resistance can lead to an increased risk of virological failure and modelling predicts that increased TDR would have a significant impact on future HIV mortality [3–5]. Onward transmission of antiretroviral drug resistance from patients failing first-line therapy may therefore compromise South Africa’s success toward reaching the last 90% of the UNAIDS 90-90-90 target by 2020, which is achieving 90% viral suppression in patients on antiretroviral combination therapy [6].

The definition of TDR can theoretically only be applied to recently-infected individuals as transmitted mutations may revert back to wild type, or only be present at levels below the sensitivity of standard genotyping assays [7]. Since it is very challenging to identify recently-infected individuals, both recently- and chronically-infected individuals who start ART are often included in surveys.

The WHO has classified levels of TDR into three categories: low (<5%), moderate (5–15%) and high levels (>15%) [8]. The 2012 WHO HIV drug resistance report, which included survey data from 2004 to 2010, stated that most surveys (72.2%, 52/72) detected low prevalence (<5%) of TDR to all drug classes. However, 20 surveys showed a moderate level of TDR (5–15%) to one or more antiretroviral drug classes. Moderate levels of TDR were most commonly observed against the NNRTI drug class. Overall, 13 out of 20 surveys (65%) showing a moderate prevalence of TDR to any drug class were conducted in the African region, particularly east Africa [9]. A more recent meta-analysis suggested that the median overall level of TDR in sub-Saharan Africa was limited to 2.9% with a yearly 1.09-fold (95% CI: 1.05–1.14) increase in odds of TDR since national ARV scale-up, attributable to an increase in non-nucleoside reverse transcriptase inhibitor (NNRTI) resistance [10]. Earlier mathematical models have suggested that the 5% threshold for TDR would only be breached 10 years after large-scale ART roll-out, or when at least 30% of HIV-infected patients are on ART [8]. As 2014 marked a decade of large-scale ART roll-out in South Africa and more than 80% of the HIV-infected South African individuals in need of ART, based on a CD4-count of 350 cells/mm^3^ were on treatment by the end of 2012, an increasing level of transmitted and/or pre-treatment drug resistance in the South African population can be expected.

Since 2002, various surveys addressing the level of transmitted or pre-treatment drug resistance have been conducted in South Africa. For ease of reference no distinction was made between studies that targeted only recently-infected individuals versus any patient prior to ART initiation. Gauteng and KwaZulu-Natal were the most surveyed regions. The studies conducted in Gauteng, Free State and Mpumalanga consistently detected a low prevalence of TDR [11–14]. The detected prevalence in KwaZulu-Natal changed from low levels in 2005 and 2007 to moderate levels of TDR in 2008 and 2009 [13]. These moderate levels were confirmed by one study [15], but not by two additional studies from the region [16, 17]. A recent temporal analysis of transmitted drug resistance in KwaZulu-Natal showed an increase between 2010 and 2012 [18]. A single study from Limpopo also detected moderate levels of TDR [19]. Despite the prevalence of TDR remaining low in the Western Cape, the latest data suggest that this prevalence is on the rise [20–22].

This study therefore aimed to conduct the very first national pre-treatment drug resistance survey to assess the current situation in the whole of South Africa.

## Materials and Methods

### Survey design

In estimating the proportion of ART naïve patients that had transmitted or pre-treatment resistance at a national level, we had two considerations: the size of the sample and how to select samples to represent South Africa. For the size of the sample, with 277 sequences we had sufficient statistical power to detect a 10% prevalence with a margin of error of +/- 3.5%, which we deemed sufficient to estimate this proportion. We selected the samples from the provinces proportional to the HIV population of the sample in order to achieve a proportional distribution of ART naïve individuals using probability proportional to size sampling (PPSS). South Africa has 9 provinces and 52 districts. Only tier two facilities with at least 500 patients in ART care were considered, to ensure adequate sampling options. The number of patients in care per facility was obtained from the South African District Health Information System. Any patient initiating ART or attending a pre-treatment follow-up visit, without prior ART exposure, who was at least 18 years of age and had signed informed consent, was eligible to be included in the study. Due to the lack of research staff at the health care facilities, a non-random sample of patients was used for this survey.

### Data and sample collection

Health care professionals at sentinel sites informed eligible patients of the HIV antiretroviral drug resistance survey. After obtaining written informed consent, a study questionnaire was completed. The study questionnaire included questions regarding basic demographics, ART history and CD4 counts. Subsequently, whole blood was collected in EDTA-vacutainers. The samples were then sent to one of three participating HIV genotyping laboratories: Tygerberg Hospital, Stellenbosch processed samples collected in the Western Cape; Universitas, Bloemfontein, processed samples collected in the Free State and the laboratory at Charlotte Maxeke Johannesburg Academic Hospital, Johannesburg processed the samples collected in the remaining provinces. To ensure sample integrity, it was advised that the specimens should reach the relevant laboratories within 72 hours after sample collection. Upon receipt, samples were centrifuged and plasma was stored at -80°C until processing. Each laboratory used their own validated population-based in-house genotyping method to obtain *pol* sequences. The in-house genotyping assay used at Tygerberg hospital covers protease (PR) amino acid (aa) 1 to 99 and reverse transcriptase (RT) aa 1 to 262 and has a lower limit of detection of 400 copies/ml [23] The in-house genotyping assay used at Universitas covers PR aa 1 to 99 and RT aa 1 to 240 and has a lower limit of detection of 1000 copies/ml. Finally, the in-house genotyping assay used at Charlotte Maxeke Johannesburg Academic Hospital covers PR aa 1 to 99 and RT aa 1 to 418 and has a lower limit of detection of 1000 copies/ml [24, 25].

### Sequence analysis

All nucleotide sequences obtained were collated at Charlotte Maxeke Johannesburg Academic Hospital for analysis. In addition, the Stanford CPR v6.0 (Calibrated Population Resistance) tool (http://cpr.stanford.edu/cpr.cgi) was used to interpret the level of pre-treatment drug resistance in the ART naïve population. This tool is based on the 2009 WHO surveillance drug resistance mutation (SDRM) list [26]. The Stanford HIVdb v7.0.1 tool (http://sierra2.stanford.edu/sierra/servlet/JSierra) was used to generate predicted resistance profiles. Predicted antiretroviral drug resistance profiles were categorised as either being susceptible (including potential low-level resistance), intermediate (including low-level resistance) or high-level resistance.

### Subtyping and cross-contamination check

A multiple alignment of the 277 nucleotide sequences with the 2010 subtype reference sequences from HIV-1 subtypes A to K, and several CRFs (http://hiv-web.lanl.gov), were generated using Clustal X (version 2.1) (http://www.clustal.org) to determine subtypes as well as confirm there was no cross-contamination amongst samples. The generated Clustal X alignment was used to construct a Neighbor-Joining phylogenetic tree in MEGA 7 (http://www.megasoftware.net) with the Kimura two-parameter model. The stability of the nodes was assessed by bootstrap analysis (1000 replicates), and bootstrap values greater than 70% were considered significant. An additional phylogenetic analysis was conducted for all sequences with at least one SDRM, to exclude clustering among resistant sequences.

In addition *pol* subtyping was confirmed by using the Rega HIV subtyping tool v2.0 (http://bioafrica.net/rega-genotype/html/subtypinghiv.html). Any sequence that was assigned to be a non-C subtype was also analysed using the NCBI genotyping tool (http://www.ncbi.nlm.nih.gov/projects/genotyping/) and the jumping profile HMM tool (http://jphmm.gobics.de/submission_hiv.html) to confirm the subtype.

### Statistical analysis

Statistical analyses were performed using GraphPad Prism 6. For all prevalence calculations, 95% confidence intervals were calculated using the modified Wald Method.

### Sequence Data

The *pol* nucleotide sequences were submitted to GenBank using Sequin v13.70 (www.ncbi.nlm.nih.gov/Sequin) and are available under accession numbers KT892975-KT893251.

### Ethics Statement

This study was conducted with ethical clearance by the Research on Human Subjects (Medical) Committee at the University of the Witwatersrand (Clearance Number M120254). Written informed consent was obtained from all participants prior to sample collection.

## Results

Patient samples were collected between March 2013 and October 2014. Only 320 samples out of the originally targeted 336 samples were collected. Samples from North West and Western Cape provinces were underrepresented due to the inability to motivate health care workers and/or patients to participate in the survey. For 43 samples, sequences could not be included due to the inability to obtain a PCR product (n = 31), inadequate completion of the consent form (n = 5) or participants being younger than 18 years (n = 7). Finally, a total of 277 sequences were available across all nine provinces (Table 1, S1 Fig). The median age of participants was 34 years (IQR: 29–42) and most participants were female (n = 163, 58.8%). Self-reported time of diagnosis of HIV-positive status was available for 258 participants at a median of 44 days (IQR: 23–179) prior to sample collection. Baseline CD4 counts were available for 195 participants and were obtained 16 days (median) before the date of sample collection (IQR: 8–36). The median baseline CD4 count was 149 cells/mm^3^ (IQR: 62–249).

**Table 1:** Prevalence of surveillance drug resistance mutations (SDRM) amongst HIV-1 infected individuals throughout South Africa.

Province	Patients on ART (%)	Assigned samples (n)	Available sequences (n)	Any SDRM (n)	Any SDRM (%)	Any SDRM (95% CI)	NNRTI SDRM (n)	NNRTI SDRM (%)	NNRTI SDRM (95% CI)	NRTI SDRM (n)	NRTI SDRM (%)	NRTI SDRM (95% CI)	PI SDRM(n)	PI SDRM(%)	PI SDRM(95% CI)
Eastern Cape	7.8	26	25	4	16.0	5.8–35.3	4	16.0	5.8–35.3	1	4.0	0.0–21.1	0	0.0	0.0–15.8
Free State*	7.6	25	25	3	12.0	3.3-30-8	3	12.0	3.3-30-8	1	4.0	0.0–21.1	0	0.0	0.0–15.8
Gauteng	15.8	53	47	3	6.4	1.6–17.8	3	6.4	1.6–17.8	1	2.1	0.0–12.1	0	0.0	0.0–9.0
KwaZulu-Natal**	38.3	129	121	11	9.1	5.0–15.7	10	8.3	4.4–14.7	3	2.5	0.5–7.4	1	0.8	0.0–5.0
Limpopo	4.7	16	13	1	7.7	0.0–35.4	1	7.7	0.0-35-4	0	0.0	0.0–26.6	0	0.0	0.0–26.6
Mpumalanga*	8.1	27	27	2	7.4	1.0–24.5	1	3.7	0.0–19.8	1	3.7	0.0–19.8	1	3.7	0.0–19.8
North West	10.6	36	13	1	7.7	0.0–35.4	1	7.7	0.0–35.4	0	0.0	0.0–26.6	0	0.0	0.0–26.6
Northern Cape	0.4	1	1	0	0.0	0.0–83.3	0	0.0	0.0–83.3	0	0.0	0.0–83.3	0	0.0	0.0–83.3
Western Cape	6.7	23	5	0	0.0	0.0–48.9	0	0.0	0.0–48.9	0	0.0	0.0–48.9	0	0.0	0.0–48.9
**TOTAL**	**100.0**	**336**	**277**	**25**	**9.0**	**6.1–13.0**	**23**	**8.3**	**5.6–12.2**	**7**	**2.5**	**1.1–5.2**	**2**	**0.7**	**0.0–2.8**

* 1 sample with ≥ 4 mutations

** 2 samples with ≥ 4 mutations

The most common *pol* subtype was HIV-1 subtype C (98.6%). Five sequences belonged to non-C subtypes. One patient from the Eastern Cape was infected with HIV-1 subtype D; two patients from Gauteng were infected with HIV-1 subtype B and CRF02_AG, respectively. The last non-C subtype was detected in KwaZulu-Natal (recombinant between subtype C and A2).

Overall, 25 out of 277 surveyed patients presented with at least one antiretroviral drug resistance mutation. This finding translates into an overall moderate pre-treatment HIV-1 resistance prevalence of 9.0% (95% CI 6.1–13.0%, Table 1). Phylogenetic analysis of these 25 sequences did not reveal any clustering (S2 Fig).NNRTI mutations contributed to the majority of detected mutations, with 23 patients presenting with at least one NNRTI mutation (8.3%; 95% CI 5.6–12.2%). The most common NNRTI mutation was K103N (n = 16) followed by Y181C (n = 6) and V106M (n = 3). Seven (2.5%) participants presented with at least one NRTI mutation. The K65R mutation was the most frequently detected NRTI mutation (n = 4) followed by M184V (n = 3). Six individuals (2.2%) were infected with a virus harbouring a combination of at least one NNRTI and one NRTI mutation. Only two participants (0.7%, 95% CI 0.0–2.8%) presented with a PI mutation.

The presence of at least four mutations was observed in four patients. All of these patients presented with the M184V and/or the K65R mutation. Details about the SDRM detected in the viruses from these 25 patients are provided in Table 2.

**Table 2:** Detailed antiretroviral drug resistance mutation profiles detected in 25 of the 277 pre-treatment patient samples.

Sample ID	# NNRTI SDRMs	NNRTI SDRMs	# NRTI SDRMs	NRTI SDRMs	# PI SDRMs	PI SDRMs
GF-EC-A-013	1	K103N	None		None	
GF-EC-A-015	1	K103N	None		None	
GF-EC-A-020	1	Y188L	None		None	
GF-EC-A-018	1	G190S	1	M184V	None	
GF-FS-A-001	1	K103N	None		None	
GF-FS-A-012	1	L100I	None		None	
GF-FS-A-007	3	K103N, V106M, Y181C	2	K65R, D67N	None	
GF-GP-A-049	1	K103N	None		None	
GF-GP-A-034	1	K103N	None		None	
GF-GP-A-021	1	Y181C	1	K65R	None	
GF-KZ-A-003	1	V106M	None		None	
GF-KZ-A-035	1	K103N	None		None	
GF-KZ-A-057	1	K103N	None		None	
GF-KZ-A-063	1	K103N	None		None	
GF-KZ-A-089	1	K103N	None		None	
GF-KZ-A-099	1	K103N	None		None	
GF-KZ-A-092	2	K103N, Y181C	None		None	
GF-KZ-A-111	2	K103N, P225H	None		None	
GF-KZ-A-073	None		1	L210W	1	L90M
GF-KZ-A-014	3	K103N, V106M, M230L	1	K65R	None	
GF-KZ-A-051	3	K101E, Y181C, G190A	2	K65R, M184V	None	
GF-LP-A-011	1	Y181C	None		None	
GF-MP-A-020	2	K103N, Y181C	6	M41L, Y115F, F116Y, Q151M, M184V, T215Y	None	
GF-MP-A-009	None		None		1	V32I
GF-NW-A-003	1	K103N	None		None	

Samples highlighted in grey might not represent true ART naïve patients.

Although no data on the regimen initiation after sample collection was obtained, an assumption has been made that all patients included in the survey would be initiated on the standard first-line regimen comprising a combination of tenofovir (TDF), lamivudine/emtricitabine (3TC/FTC) and efavirenz (EFV) as per national guidelines [27]. Results of the sequence analyses imply that 91.0% (n = 252) of these patients, without any detected antiretroviral drug resistance, would be initiated on a fully-active triple ART combination. In two additional patients (0.7%), the detection of a resistance mutation would not affect the activity of the recommended first-line regimen. Seventeen patients (6.1%) showed resistance against NVP and EFV prior to ART initiation, which implies that only the NRTI backbone would be active in these patients. Essentially, these patients are started on a dual regimen. Finally, one patient (0.4%) would receive TDF monotherapy, whereas for five patients (1.8%) none of the three drugs in the standard first-line regimen would be active.

## Discussion

This pre-treatment antiretroviral drug resistance survey was designed to assess the current prevalence of HIV-1 drug resistance among South African HIV-infected patients prior to ART initiation in all nine provinces for the first time. The obtained data is important to verify whether the currently recommended first-line ART regimens are still adequate to ensure a good treatment outcome in most HIV-infected patients initiating these regimens. This is the first survey which includes samples from every province in South Africa, based on the proportion of patients receiving ART in each region.

The observed pre-treatment resistance prevalence is classified as a moderate level of 9.0% (95% CI: 6.1% to 13.0%) according to the WHO classification. This level of pre-treatment drug resistance was only detected in three South African studies prior to this survey: 9.3% in Limpopo [19] and 7.4 [15] and 13.5% [13] in KwaZulu-Natal. However, the increasing prevalence over the years might be explained by the hypothesis that mature ART programmes can be a proxy for higher levels of pre-treatment drug resistance [11]. However, other factors, which were beyond the scope of this study, such as virological failure rates, adherence levels in the treated population and drug supply continuity, can also affect the levels of pre-treatment drug resistance. Earlier statistical models predicted that the 5% threshold for TDR would only be breached 10 years after large-scale ART roll-out or when at least 30% of HIV-infected patients are on ART [8]. As 2014 marks a decade of large-scale ART roll-out in South Africa and more than 80% of the HIV-infected South African individuals in need for ART were on treatment since the end of 2012, the increasing levels of transmitted and/or pre-treatment drug resistance in the South African population are not unexpected [3]. NNRTI mutations remain the most commonly observed pre-treatment resistance mutations. Non-TAM mutations such as K65R and M184V mutations were more commonly observed compared to TAMs, this is a reflection of the replacement of d4T and AZT by TDF in first-line regimens since 2010 [28]. To our knowledge it is also the first report on pre-treatment drug resistance that showed the K65R mutation to be the most commonly observed NRTI mutation.

Based on these findings, only 2.2% of the population initiating ART would likely be started on monotherapy, or a completely inactive regimen and 6.1% of the study population would be initiated on dual ART (active TDF and 3TC/FTC). Hamers *et al*. demonstrated in a large multi-centre study that pre-treatment drug resistance does have an impact on the outcome of first-line treatment with an odds ratios for increased virological failure of 2.13 (95% CI: 1.44–3.14). In addition, patients with pre-treatment drug resistance are more likely to develop acquired drug resistance as compared to those patients without pre-treatment drug resistance (OR 2.30, 95% CI: 1.55–3.40) [4].

It can be argued that the patients with multiple antiretroviral drug resistance mutations, without recorded treatment history, were either not truly drug-naïve or might have been infected with a multidrug resistant virus. Several studies, especially cluster transmission studies, have shown that resistance mutations with minimal impact on viral fitness may be transmitted serially without the need for intervening drug pressure [29, 30]. However, the presence of K65R and/or M184V in each of these individuals might indicate these patients were not truly ART naïve due to the high fitness cost of both mutations [31–33]. Treatment histories of these patients with multiple SDRMs were verified and health care workers confirmed these patients did not disclose and prior ART exposure. The resistance profile of these patients is of concern as the likelihood of virological suppression on standard first-line regimens is limited. However, health care professionals would not be able to become aware of these mutations in routine care since HIV-1 drug resistance testing is currently not recommended prior to ART initiation.

Despite the adequate sample size and PPSS study design, the study had some limitations. Firstly, the enrolment of patients into the study was largely dependent on the willingness of clinicians and nurses to obtain consent from patients, complete study questionnaires and collect specimens, as there was no provision of study-specific staff to assist in the survey. These activities place a burden on clinics that are often already under-resourced and thus demonstrate the difficulties in conducting such surveys and the vast costs associated with their completion. These challenges resulted in a relatively long period to complete sample collection. Overall 82% of the planned sample size was collected and analysed. But there was under-sampling from North West, and Western Cape provinces.

Secondly, patients were not systematically sampled at the clinic level. This approach might have introduced a selection bias as sample selection was dependent on willingness of health care workers and patients to comply with the added burden of the study. However, we don’t believe that it is possible for clinicians to choose treatment naïve patients based upon demographic or clinical characteristics that would bias the proportion of SDRM.

Thirdly, data quality was dependant on the completeness and accuracy of the questionnaires. Moreover, no specific information regarding exposure to Prevention-of-Mother-to-Child-Transmission prophylaxis was obtained and may have potentially overestimated the level of pre-treatment drug resistance caused by the use of PMTCT regimens, especially since most patients were female. In addition, information regarding prior ART exposure could only be verified based on verbal confirmation from participants or notes in the patient’s hospital files, which might have led to the inclusion of patients with undisclosed ART exposure. Finally, resistance mutations were detected by population-based Sanger sequencing which might underestimate the prevalence of minority drug resistance mutations, due to sensitivity limitations of this approach, especially in patients who have been chronically infected before ART initiation. This study might have underestimated the level of pre-treatment resistance; since it is likely the sample included a substantial proportion of chronically infected patients, indicated by the low median CD4 count of 149 cells/mm^3^. It is known that TDR mutations can revert back to wild type in the bulk viral population, especially without the presence of drug pressure. It is therefore possible that archived mutations were missed in this analysis. However, these limitations are largely overcome by the large sample size of the study and the fact that samples were collected from all provinces, proportional to the number of patients on ART.

Overall, this study has shown that the level of antiretroviral drug resistance in ART-naïve South Africans is on the rise. Regional analyses indicate that there might be a difference in the level of drug resistance between provinces, although this trend needs to be confirmed by repeating surveys in each province with larger sample sizes. Despite the availability of routine viral load testing in the public sector, there is often a delayed response to changing management based on virological failure which could have contributed to the transmission of drug resistance [34]. These findings strongly suggest a renewed focus on first-line treatment success (the last 90% of the UNAIDS 90-90-90 strategy) with regular regional surveillance of pre-treatment drug resistance levels in all regions of the country.

## Supporting Information

S1 Fig Geographical map of South African indicating the health care facilities within the 9 provinces that contributed samples to the ART-naïve survey. Each circle indicates a health care facility that contributed specimens to the survey. The size of the circles is proportional to the number of specimens collected at each health care facility. The grey scale indicates the number of patients on ART in each province.
(DOCX)

S2 Fig Phylogenetic tree including all 25 sequences with at least one SDRM. No clustering is observed among these samples.
(DOCX)
