# Supplementary material for: Moderate Levels of Pre-Treatment HIV-1 Antiretroviral Drug Resistance Detected in the First South African National Survey
Source: PLoS One. 2016 Dec 1;11(12):e0166305. doi: 10.1371/journal.pone.0166305 (PMC5132262; doi:10.1371/journal.pone.0166305)

Supplementary Figure 1: Geographical map of South African indicating the health care facilities within the 9 provinces that contributed samples to the ART-naïve survey.

Each circle indicates a health care facility that contributed specimens to the survey. The size of the circles is proportional to the number of specimens collected at each health care facility. The grey scale indicates the number of patients on ART in each province.


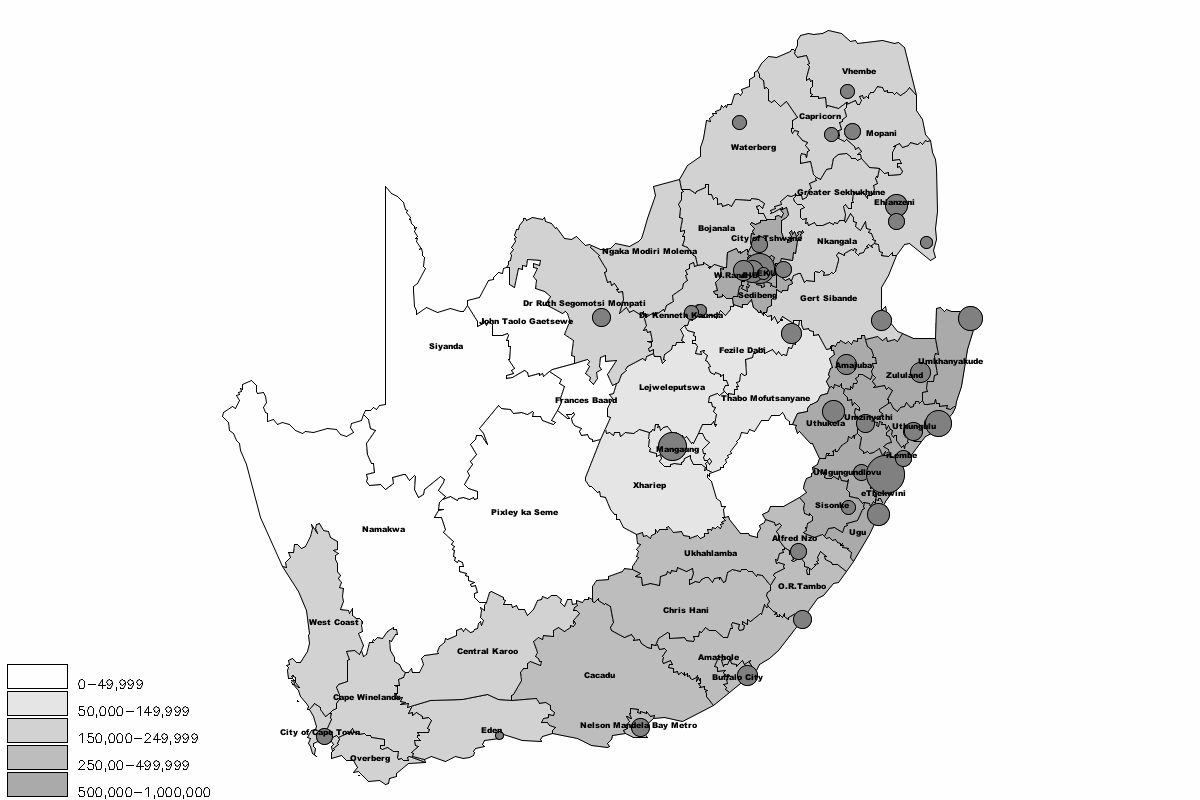

Supplement: S1 Fig — Each circle indicates a health care facility that contributed specimens to the survey. The size of the circles is proportional to the number of specimens collected at each health care facility. The grey scale indicates the number of patients on ART in each province. (DOCX) [file pone.0166305.s001.docx]
