# Supplementary material for: Moderate Levels of Pre-Treatment HIV-1 Antiretroviral Drug Resistance Detected in the First South African National Survey
Source: PLoS One. 2016 Dec 1;11(12):e0166305. doi: 10.1371/journal.pone.0166305 (PMC5132262; doi:10.1371/journal.pone.0166305)
Supplement: S2 Fig — No clustering is observed among these samples. (DOCX) [file pone.0166305.s002.docx]

Supplementary Figure 2: Phylogenetic tree including all 25 sequences with at least one SDRM. No clustering is observed among these samples.
